# Supplementary material for: Refining CT image analysis: Exploring adaptive fusion in U-nets for enhanced brain tissue segmentation
Source: PLoS One. 2025 Jun 11;20(6):e0323692. doi: 10.1371/journal.pone.0323692 (PMC12157114; doi:10.1371/journal.pone.0323692)
Supplement: S1 Appendix — (DOCX) [file pone.0323692.s002.docx]

**Appendix**

**Investigation into the generalization capability of the proposed adaptive fusion strategy**

To assess the generalizability of our study design and findings, we utilized 4298 cat images from the COCO 2017 dataset, an example of which is depicted in S1 Figure, to conduct the segmentation experiment. As illustrated in Figure 5, UNet3+ exhibited the most favorable performance for brain tissue segmentation, followed by UNet2+ and U-Net. Our aim is to determine whether a similar outcome can be observed with the cat dataset.

The segmentation experiments outlined in Tables 1 and 2 were replicated, and the resulting performances are presented in Tables 5 and 6. These results are consistent with our previous analysis using NCCT images. Notably, upon comparing the results generated solely by each of the three networks, UNet3+ consistently displayed the best performance. The application of filtering appears to offer limited improvement, as the target (i.e., cat) typically exhibits clear edges against the background, and the segmentation models do not generate significant false alarms compared to brain CT data.

Furthermore, we conducted result fusion of U-Net/UNet2+ and UNet2+/UNet3+, reporting the best outcome across various optimizer and learning rate settings for each fusion in S1 Table. The fusion of UNet2+ with UNet3+ consistently yielded the highest IoU and the lowest HD. S1 Figure illustrates an example image alongside its corresponding ground truth mask, while S2 Figure presents the segmentation results as follows: (a) Origat image; (b) Corresponding ground truth image; (c) Segmentation outcome using U-Net; (d) Segmentation outcome using UNet2+; (e) Segmentation outcome using UNet3+; (f) Fusion result optimized by combining UNet2+ & UNet3+.

**S1 Figure.** Example image (left) and corresponding ground truth mask (right).

**S2 Figure.** Illustration of segmentation results. (a) Original cat image; (b) Ground truth image; (c) Segmentation result using U-Net; (d) Segmentation result using UNet2+; (e) Segmentation result using UNet3+; (f) Optimal fusion result (UNet2+ & UNet3+).

**S1 Table.** Result fusion with UNet2+ (optimizer=Adagrad, learning rate=0.0001). The best-performing results are highlighted using bold font.

| Model  (optimizer, learning rate) | Prediction | | Applied filtering | | |
| --- | --- | --- | --- | --- | --- |
|  | IoU | HD | | IoU | HD |
| U-Net  (Adagrad, 0.0001) | 0.385 | 1963.5 | | 0.387 | 2003.8 |
| UNet3+  (Adam, 0.001) | 0.461 | **584.1** | | **0.463** | 590.9 |
| Fusion all | 0.450 | 728.0 | | 0.453 | 749.3 |
