# Supplementary material for: Refining CT image analysis: Exploring adaptive fusion in U-nets for enhanced brain tissue segmentation
Source: PLoS One. 2025 Jun 11;20(6):e0323692. doi: 10.1371/journal.pone.0323692 (PMC12157114; doi:10.1371/journal.pone.0323692)
Supplement: S1 Table — The best-performing results are highlighted using bold font. (DOCX) [file pone.0323692.s003.docx]

Result fusion with UNet2+ (optimizer=Adagrad, learning rate=0.0001). The best-performing results are highlighted using bold font.

| Model  (optimizer, learning rate) | Prediction | | Applied filtering | | |
| --- | --- | --- | --- | --- | --- |
|  | IoU | HD | | IoU | HD |
| U-Net  (Adagrad, 0.0001) | 0.385 | 1963.5 | | 0.387 | 2003.8 |
| UNet3+  (Adam, 0.001) | 0.461 | **584.1** | | **0.463** | 590.9 |
| Fusion all | 0.450 | 728.0 | | 0.453 | 749.3 |
