# Supplementary figures and images for: Refining CT image analysis: Exploring adaptive fusion in U-nets for enhanced brain tissue segmentation
Source: PLoS One. 2025 Jun 11;20(6):e0323692. doi: 10.1371/journal.pone.0323692 (PMC12157114; doi:10.1371/journal.pone.0323692)

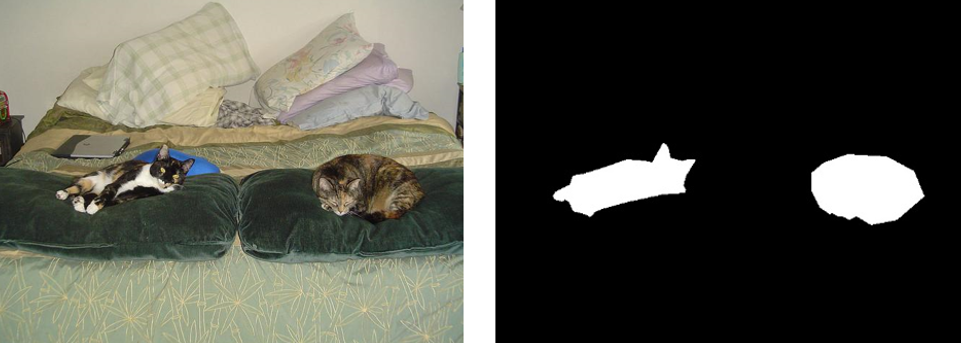

Supplement: S1 Fig — (TIF) [file pone.0323692.s004.tif]

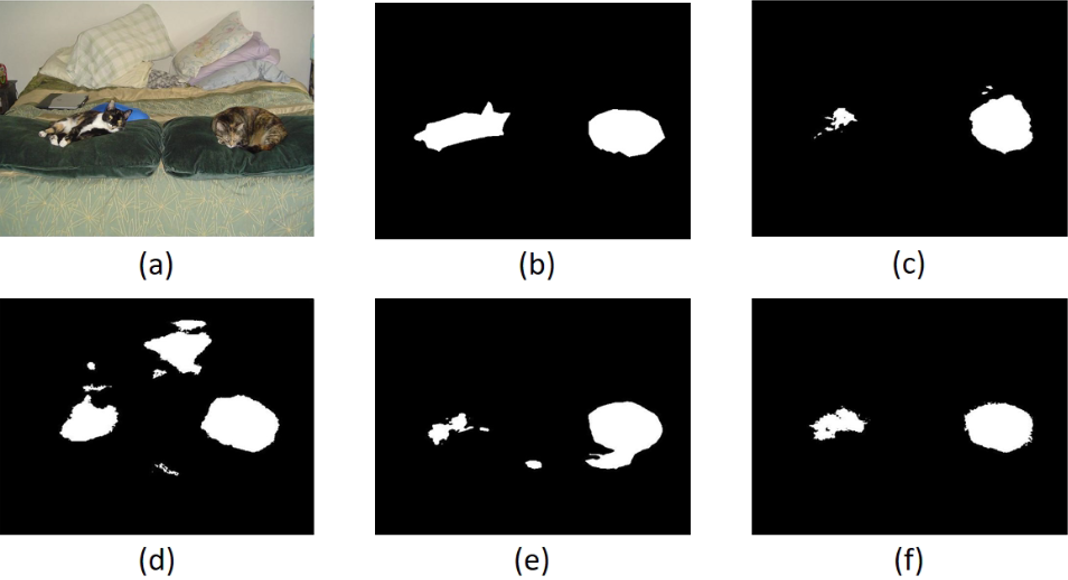

Supplement: S2 Fig — (a) Original cat image; (b) Ground truth image; (c) Segmentation result using U-Net; (d) Segmentation result using UNet2 + ; (e) Segmentation result using UNet3 + ; (f) Optimal fusion result (UNet2 + & UNet3+). (TIF) [file pone.0323692.s005.tif]
